# Drought stress induces early flowering and the stress tolerance of offspring in *Petunia hybrida*

**DOI:** 10.5511/plantbiotechnology.23.1220a

**Published:** 2024-03-25

**Authors:** Ngoc-Ha Thi Tran, Duong Van Hoang, Loc Tuong Phan

**Affiliations:** 1 Institute of Tropical Biology, Vietnam Academy of Science and Technology, Linh Trung, Thu Duc, Ho Chi Minh City 700000, Vietnam

**Keywords:** drought stress, early flowering, *Petunia hybrida*, seedling vigor, stress memory

## Abstract

*Petunia hybrida* (Solanaceae) exhibits high sensitivity to water scarcity, especially during flowering. This study investigated changes in the flowering time of *P. hybrida* in response to water deficit over a 7-week period. Various levels of water stress—i.e., light, moderate, and severe—were imposed on plants grown in a greenhouse, and these were compared to a control group grown alongside. Remarkably, early flowering was observed under severe stress in *P. hybrida* for the first time, occurring 5.3 days earlier than in the control group. Furthermore, seeds collected from control and treatment plants were then used to assess drought stress memory in offspring. Seedlings were cultivated in a dehydration medium containing either PEG 8000 or a control MS medium. In the PEG 8000 medium, seedlings from parents exposed to moderate and severe drought stresses exhibited higher drought tolerance than those from well-watered conditions. Moreover, they also displayed significantly longer roots, more leaves, and a lower ion leakage rate. Taken together, these findings demonstrated the presence of positive transgenerational effects on progeny. Thus, while parental drought stress during reproduction stage may affect seed quality, it can enhance drought tolerance in the next generation via induction of stress memory.

## Introduction

Plants generally respond to drought stress by delaying flowering and reducing growth to adapt to limited energy availability (Chirivì and Betti 2023; Toscano et al. 2019). In a different strategy, drought-stressed plants induce early flowering to complete their life cycle and overcome unfavorable environmental conditions (Franks 2011; Sivakumar and Srividhya 2016). In both cases, to survive stress the transition from the vegetative to the reproductive growth phase needs to be tightly controlled (Takeno 2016). Moreover, understanding how drought stress can change flowering time is necessary for establishing breeding strategies. Studies of drought stress on plant flowering and reproduction have often been performed in model plants such as *Arabidopsis* (Riboni et al. 2013) or in crops such as rice (Du et al. 2018; Kumar et al. 2023), wheat (Shavrukov et al. 2017), and tomato (Chong et al. 2022).

Moreover, parents commonly transfer stress responses to their offspring via diverse mechanisms. Some studies have demonstrated that prior exposure to mild abiotic stress can enhance a plant’s ability to adapt to subsequent episodes of the same stress (Mozgova et al. 2019). This phenomenon has been referred to as “hardening”, “acclimation”, or “plant priming”, and relies on plant stress memory (Liu et al. 2022c). Somatic memory, or mitotic stress memory, occurs when stress is encountered at the early vegetative stage and is activated later in the same generation, typically transiently (Crisp et al. 2016). In contrast, transgenerational memory (also known as meiotic stress memory) occurs when parents experienced stress during their reproductive phase, especially during seed formation. This type of memory can be passed from parents to first-generation progeny and even to subsequent generations (Liu et al. 2022a). In the past, studies of drought stress memory have focused on crops due to the importance of implications for food sustainability (Liu et al. 2022a).

*Petunia hybrida*, a member of the Solanaceae family, ranks among the most beloved ornamental crops throughout the world. In the United States, it is one of the leading ornamental bedding plants, with a wholesale value estimated at $142 million in 2018 (USDA 2019). However, *P. hybrida* exhibits sensitivity to water scarcity. Moreover, research on the relationship between flowering time and drought stress in *Petunia* remains limited (Immink et al. 1999; Tsukamoto et al. 2016). In addition, research on stress memory in ornamental plants under abiotic stress conditions is also lacking. Therefore, in this study we exposed *Petunia* plants to varying levels of water scarcity stress within a controlled greenhouse environment, spanning from the pre-reproductive stage to the reproductive phase. We did so to examine changes in flowering time—as well as other vegetative and reproductive characteristics—that were induced by drought stress. Subsequently, seeds from plants subjected to different drought stress treatments were collected and sown in a dehydration medium to investigate whether there was evidence of transgenerational memory in the offspring.

## Materials and methods

### Plant materials and cultivation conditions

Commercial seeds of *Petunia hybrida* (var. FPET019) were supplied by Floralseed Co., Ltd. (Ho Chi Minh City, Vietnam). This F1 cultivar grows best at temperatures between 25°C and 30°C. All seeds were sown in a square plastic tray (i.e., 5 cm width and 5 cm depth) filled with peat moss, Klasmann TS2 (Klasmann-Deilmann, Geeste, Germany). This trial was performed in a greenhouse located in Thu Duc City, Ho Chi Minh City, Vietnam, from October 2021 to February 2022. After 4 weeks, each seedling (i.e., at the five true leaf stage) was transferred to a vinyl pot (i.e., 12 cm diameter, 10 cm depth) filled with a mixture of Tribat® soil, perlite, and red granular (3 : 1 : 1). The temperature and humidity in the greenhouse were recorded by a data logger (model GSP-6, Elitech, London, UK).

### Water deficit assay

All plants were watered by drip irrigation at 100% pot capacity (PC) every morning. The determination of pot capacity (also called field capacity) followed a method described in a previous study (Steadman et al. 2004). Five weeks following the transplanting of the seedlings into vinyl pots, we conducted water deficit assessments. Drought stress was induced using three distinct irrigation levels, corresponding to 75%, 50%, and 25% of PC, representing light, moderate, and severe stress conditions, respectively. Additionally, a control group was included with a watering level set at 100% PC. Each treatment group consisted of 40 pots. The drought assay lasted for seven weeks, and was followed by a two-week recovery period during which all pots received irrigation at 100% PC. Soil moisture measurements were taken at a depth of approximately 5 cm from the surface using a soil moisture meter (model DM-18, Takemura Electric, Nara, Japan). Ten pots of each treatment were randomly measured for soil moisture measurements three times per week.

### Chlorophyll index and the maximum quantum efficiency of photosystem II

Leaf chlorophyll index (Chl) was assessed using a portable chlorophyll content meter (model CL-01, Hansatech, Kings Lynn, UK). The maximum quantum efficiency of photosystem II (Fv/Fm) in dark-adapted leaves was determined using a pulse amplitude modulated fluorimeter (model FSM-2, Hansatech, Kings Lynn, UK). Chl and Fv/Fm were measured using the same leaf samples; these samples were taken from 20 plants of each treatment group. For each individual plant, three points per leaf were evaluated and the average of the three values was calculated. Data on these two characteristics were recorded every 2 weeks (Supplementary Table S1).

### Plant growth rate

Plant height was recorded bi-weekly (Supplementary Table S1). The main stem height was measured from the soil surface to the tip of the shoot, and branch height was measured from its node to the top shoot. The plant’s overall height was calculated as the sum of the main stem and branch heights. To calculate the relative growth rate (RGR), the following formula was applied (Handayani and Watanabe 2020). 
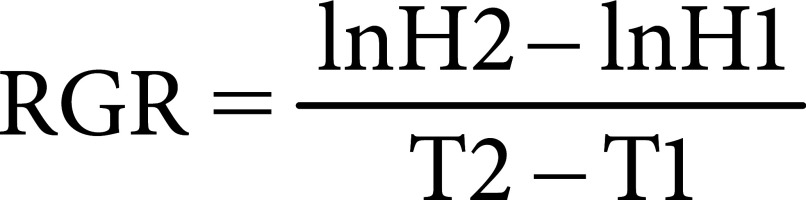
 Here, H1 represents plant height at Time 1 (T1) and H2 represents plant height at Time 2 (T2).

### Stomatal traits

Measurements of stomatal traits were conducted at the end of drought stress treatment (Supplementary Table S1). Stomatal impressions were obtained by applying clear nail polish to the abaxial side of the leaf. Next, dried nail polish was gently lifted off and affixed to a glass surface. Observations were performed using Olympus Digital Microscopes (model DSX1000, Olympus Corporation, Tokyo, Japan) at 40× magnification (DSX10-XLOB 40X objective lens) and measurements were made using the DSX1000 application (Olympus). Measured stomatal traits included stomata length, stomata width, stomata pore length, and stomata pore width.

### Relative water content

Leaf relative water content (RWC) was measured at the end of the treatment period (Supplementary Table S1) using the method described by Arndt et al. (2015). In brief, the third or fourth leaf from the shoot was collected from 12 plants of each treatment and weighed immediately to calculate the fresh weight (FW). Then, each leaf was soaked in distilled water upright with its petiole and was maintained at 10°C for 2 h. Following immersion, excess water was removed by gently blotting the leaf on a paper towel, and the resulting weight was recorded as the turgid weight (TW). Finally, dry weight (DW) measurements were obtained by drying the leaf in an oven at 60°C for 48 h then weighing it. The RWC was calculated using the following formula (Arndt et al. 2015): 
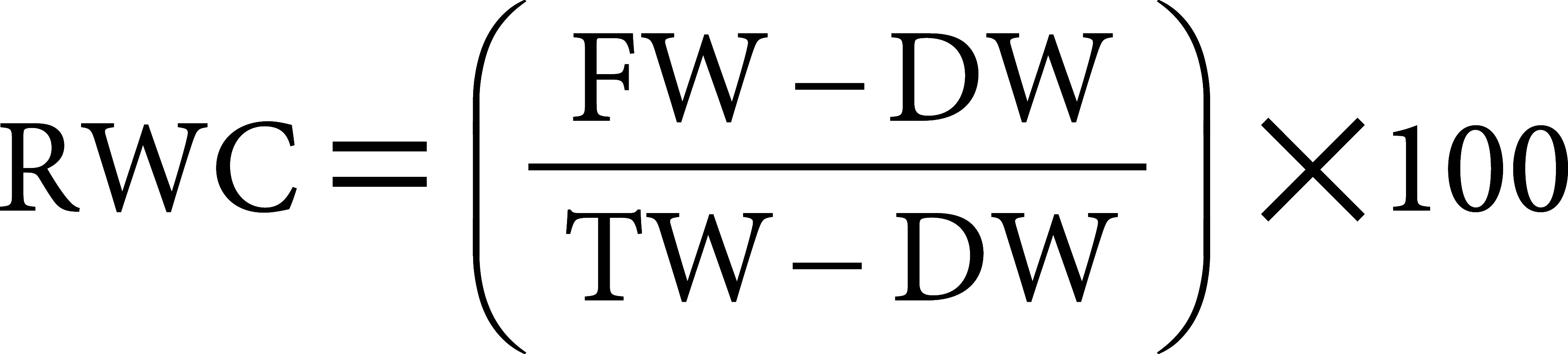


### Ion leakage rate

To assess cell membrane damage in plants throughout the drought treatment in the greenhouse, the ion leakage rate (IL) was measured by the end of the treatment (Supplementary Table S1). For each treatment, the third or fourth leaf from the shoot tip of 12 plants was selected for analysis. Three leaf discs were excised from each leaf using a cork borer (ϕ=8 mm); these were then immersed in 2 ml deionized water (Merck, Darmstadt, Germany) in a 5 ml tube and gently shaken at 60 rpm for 2 h at room temperature. Next, 100 µl of the suspension was collected from each tube, and initial conductivity (C1) values were measured using a conductivity meter (model EC22, Horiba, Fukuoka, Japan). Subsequently, the tubes were boiled for 15 min, left to cool down to room temperature, and vigorously shaken for 3 h. After shaking, another 100 µl of the suspension was collected from each tube, and final conductivity (C2) values were obtained using the conductivity meter. The IL value was calculated as (C1/C2)×100 (Chubachi et al. 2023).

To assess cell membrane damage in seedlings following 14 days of dehydration stress in polyethylene glycol 8000 (PEG 8000) medium, the leaves of all seedlings (excluding cotyledons) from each replicate of every treatment were gathered and placed in 4 ml of deionized water inside a 5 ml tube. IL was determined using the same procedure described above.

### Flowering time

The first bud time (FBT) represented the duration in days from the start of the treatment to the day when the first bud was visible. Similarly, the first flowering time (FFT) signified the number of days from the start of the treatment until the blooming of the first flower.

### Pollen quantification and viability

Pollen quality assessment was performed after four weeks of drought treatment (Supplementary Table S1) using the Alexander staining method with some modifications (Peterson et al. 2010). Briefly, anthers were harvested from at least ten flowers of each treatment for seven days; each collection was made one day prior to anthesis. Five anthers (equivalent to one flower) were promptly fixed in 150 µl of Carnoy’s fixative (Peterson et al. 2010) within 1.5 ml tubes. They were then stored at 15°C until staining. Anthers from one flower were placed in a 1.5 ml tube containing 100 µl of Alexander staining solution. Subsequently, pollen grains were gently released from the anthers using a 1.0 µl pipette tip then allowed to stand at room temperature for 30 min. The number of pollen grains was quantified using a cell counting chamber (model Neubauer improved bright-line, 0.1 mm, Hirschmann, Eberstadt, Germany), with all procedures following the manufacturer’s protocol. Finally, the observation and quantification of pollen grains were carried out using Olympus Digital Microscopes at 40× magnification.

### Pollen germination

Following four weeks of drought treatment (Supplementary Table S1), freshly mature pollen was harvested from flowers at the anthesis stage. Pollen germination was carried out using a liquid culture method, pollen grains were gently released into 1 ml of liquid culture medium (Suwińska et al. 2015). This suspension of pollen grains was then placed in sterilized petri dishes (3-cm diameter) and incubated at 30°C for approximately 6 h in the dark. Germination rates were subsequently observed and quantified using an Olympus Digital Microscope.

### Impact of dehydration stress on seed germination and seedling development

Seeds obtained from plants that were subjected to drought stress treatments (i.e., 100% PC, 75% PC, 50% PC, and 25% PC) were first sterilized. Sterilization involved immersion in 70% alcohol for 1 min then in a 2% NaClO solution for 10 min while being shaken at 100 rpm. Subsequently, seeds were rinsed five times with sterilized water then dried on filter paper. To simulate dehydration stress, seeds were sown in petri dishes containing solid MS medium (0.8% agar) with the addition of 390 g l^−1^ PEG 8000 (Fisher BioReagents™, USA) (Handayani and Watanabe 2020). In parallel, a control group was grown without PEG 8000. After seven days, the seed germination rate was recorded, with germination defined as seedlings with radicles reaching a minimum length of 5 mm.

Seeds from the four drought stress treatments were then sown on solid MS medium to evaluate seedling development. After approximately five days, seedlings with a hypocotyl of at least 1 mm were transferred to a dehydration medium as described above. Seedlings were then cultivated in a growth room maintained at a temperature of 25°C±2°C, with a photoperiod of 16 h of light followed by 8 h of darkness. After 14 days, photographs were taken, and the seedlings were removed to measure root length, shoot length, and the number of leaves, including cotyledons.

### Statistical analysis

All collected data underwent statistical analysis using R version 4.2.2 (released on October 31, 2022) and/or Microsoft Excel MSO 360 (Version 2304, Build 16327.20264) (Microsoft, Redmond, WA, USA). Analyses included various statistical methods, including Student’s *t*-tests, one-way Analyses of Variance (ANOVA), and Tukey’s Honestly Significant Difference (HSD) tests. All statistical tests were conducted using R.

## Results

### Effects of drought stress on vegetative indicators

**Figure 1:**
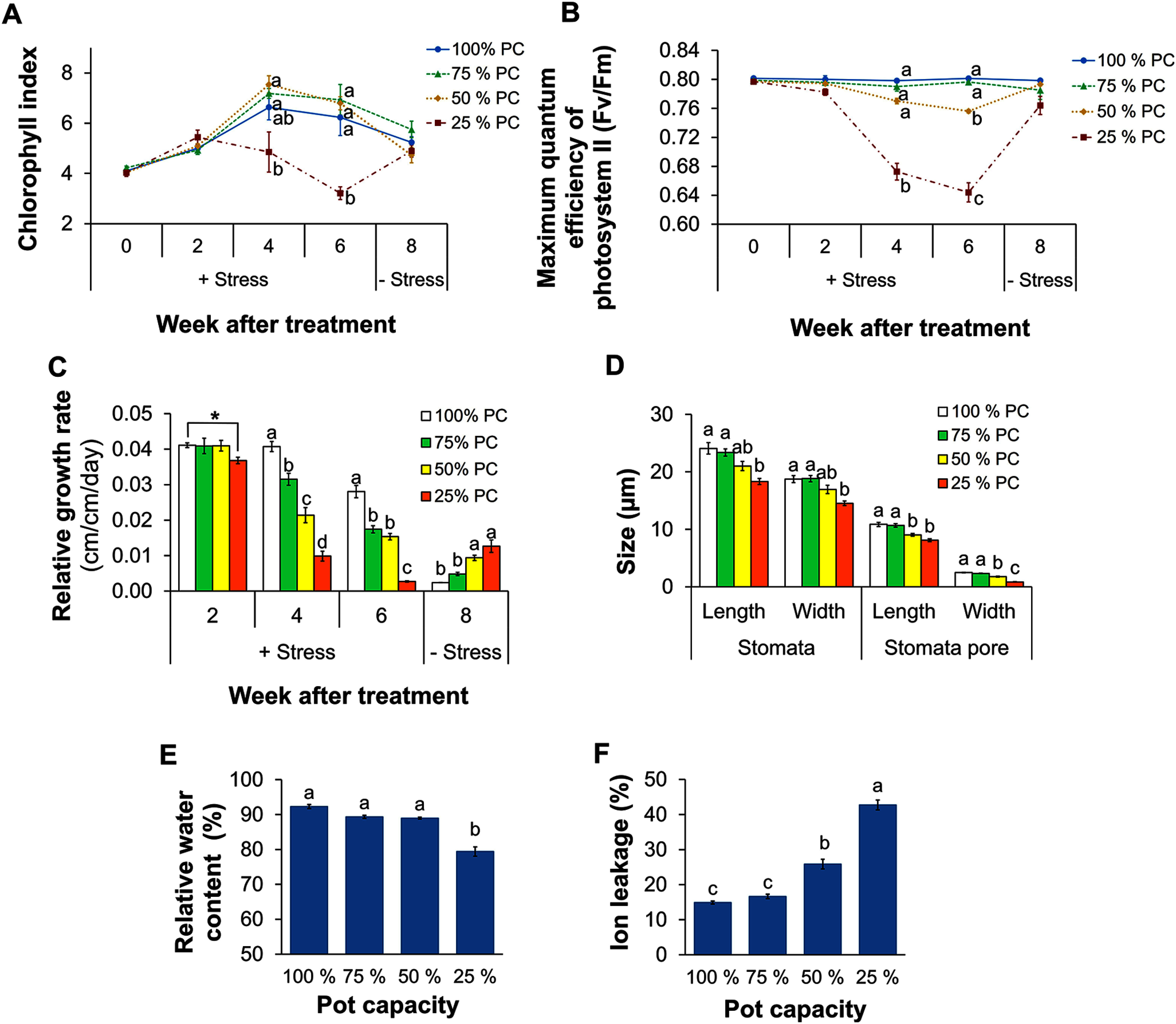
Figure 1. Effects of drought stress on vegetative indicators. (A) Chlorophyll index, (B) Maximum quantum efficiency of photosystem II (four replicates of five plants each). (C) Relative growth rate (four replications of ten plants each). (D) Stomatal traits. (E) Relative water content of leaves. (F) Ion leakage rate (four replicates of three plants each). Different letters indicate significant differences among treatments identified by Tukey-HSD tests (*p*<0.05). The asterisk sign denotes significant differences between the control (100% PC) and 25% PC treatments identified by Student’s *t*-tests; significant levels: * *p*<0.05. Error bar: Standard error. “+Stress” indicates the Drought stress period (7 weeks) with four different irrigation levels (100% PC, 75% PC, 50% PC, and 25% PC); “−Stress” indicates the Recovery period (2 weeks), after Drought stress, with 100% PC in irrigation levels.

Chl levels increased under light and moderate stress conditions (i.e., reaching 7.5) but declined significantly under severe stress (i.e., falling to 3.2) (Figure 1A). Conversely, the Fv/Fm ratio was correlated with the severity of stress (Figure 1B). Throughout the experimental period, Fv/Fm values remained approximately at 0.79 for both the control and 75% PC treatment. However, by the sixth week, values for the 50% and 25% PC treatments dropped to 0.75 and 0.64, respectively; moreover, the differences among treatments were statistically significant according to a one-way ANOVA (*p*<0.001) (Figure 1B). Following the recovery period, the Chl and Fv/Fm values across all treatments returned to levels observed in the control (Figure 1A, B). Furthermore, plant RGR decreased over the course of the experiment for all treatments (Figure 1C). However, under the severe stress of the 25% PC treatment, the RGR value experienced a dramatic decline before gradually recovering during the recovery period (Figure 1C).

Under the control conditions, the size of *Petunia* stomata measured approximately 24×19 µm. However, stomatal and stomatal pore sizes were both negatively influenced by drought stress. Significant differences were evident in both stomatal size and stomatal pore size under moderate and severe stresses (i.e., the 50% and 25% PC conditions). In contrast, the size of the stomata and stomatal pores remained nearly unchanged under low stress conditions (75% PC) (Figure 1D).

The response of *Petunia* plants to the severity of drought stress was characterized by a decrease in RWC and an increase in IL at the end of the drought stress period. The 25% PC treatment showed the lowest RWC value (79.4%), which was statistically significantly lower than the other treatments as determined by one-way ANOVA (*p*<0.001) (Figure 1E). Moreover, the 25% PC treatment exhibited the highest IL level (42.7%) followed by the 50% PC treatment (25.9%; Figure 1F).

### Impact of drought stress on reproductive characteristics

**Figure 2:**
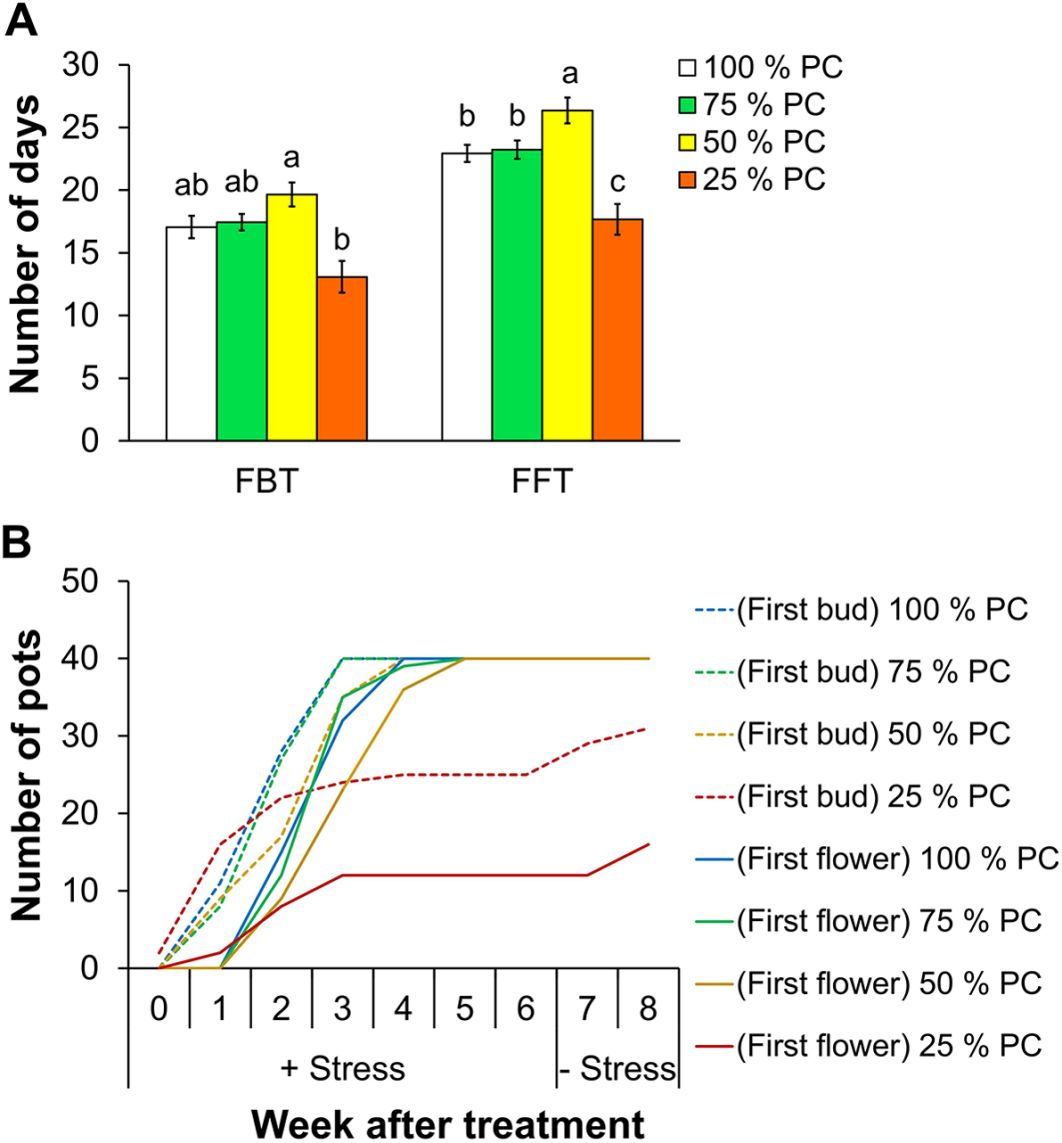
Figure 2. Flowering status under drought stress treatment. (A) Time to get the first bud and the first flower in each pot. FBT, first bud time; FFT, first flowering time. (B) Number of pots got first bud/first flower. Different letters indicate significant differences among treatments by the Tukey-HSD test (*p*<0.05). Error bar: Standard error. “+Stress” indicates the Drought stress period (7 weeks) with four different irrigation levels (100% PC, 75% PC, 50% PC, and 25% PC); “−Stress” indicates the Recovery period (2 weeks), after Drought stress, with 100% PC in irrigation levels.

In the 75% PC treatment, both the first bud time (FBT) and first flowering time (FFT) remained unaffected by decreases in soil humidity. However, under moderate drought stress (i.e., the 50% PC treatment), we observed a delay in both FBT and FFT, which occurred 2.6 days and 3.4 days later than the control group, respectively. Interestingly, this study observed early bud formation in 21 plants from the 25% PC treatment, with budding occurring four days earlier than in the control treatment. Of these 21 plants, 11 also exhibited early flowering, which occurred on average 5.3 days earlier than the control (Figure 2A). However, the remaining 10 plants subsequently shed their buds (Figure 2B). Furthermore, all flowered plants in the 25% PC treatment stopped flowering after the fourth week when the soil humidity dropped to around 12% (Figure 2B, Supplementary Figure S1). During the recovery period, only new three plants successfully flowered, nine plants did not progress to the reproductive stage, and none of the flowered plants reflowered. The appearance of representative plants from each of the four treatments before and after water deficit are shown in Supplementary Figure S2.

**Figure 3:**
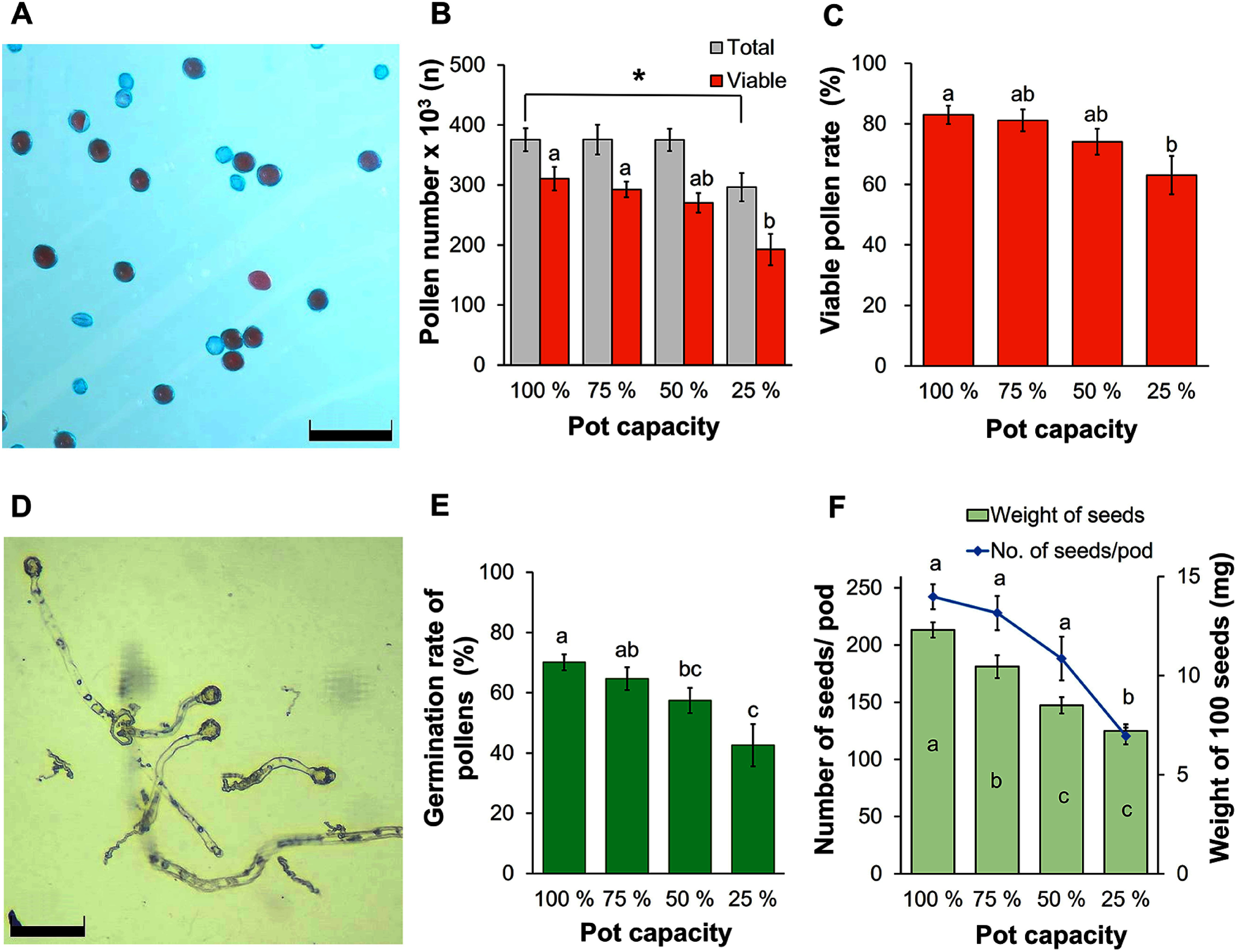
Figure 3. Pollen status under drought stress treatment and seed production. (A) Pollen staining using the Alexander staining method. Scale bar=100 µm. (B) Number of pollen per flower. Scale bar=100 µm. (C) Viable pollen rate. (D) Photo of pollen germination. (E) Germination rate of pollen. (F) Number of seeds per pod and weight of 100 seeds. Different letters indicate significant differences among treatments by the Tukey-HSD test (*p*<0.05). Asterisks denote significant differences between the control (100% PC) and 25% PC treatments identified by Student’s *t*-tests; significance level: * *p*<0.05. Error bar: Standard error.

The viable pollen rate and pollen germination rate were both assessed in the fourth week. Viable pollen grains, identifiable by their red color after staining with Alexander solution, showed an approximate diameter of 26 µm (Figure 3A). These results indicated that the number of pollen grains per flower and the viable pollen rate both remained relatively unaffected during low and moderate drought stress conditions, with values ranging from 375.3×10^3^ to 375.7×10^3^ pollen grains/flower and from 74.1% to 81.1%, respectively. However, a significant difference was observed under severe stress (i.e., 25% PC), which showed 296.5×10^3^ pollen grains/flower (significantly different from the control according to a Student’s *t*-test, *p*<0.05) and a 63.1% viable pollen rate (significantly different from other treatments according to a one-way ANOVA, *p*<0.05) (Figure 3B, C). Finally, the pollen germination rate steadily reduced in both the 50% PC and 25% PC treatments, reaching 57.4% and 42.6%, respectively. These can be compared to the control rate of 70.1% or the 75% PC treatment, which showed a slight reduction to 64.7% (Figure 3D, E).

Seed production significantly decreased in the 50% PC and 25% PC treatments, which showed 188.2 and 116.7 seeds/pod, respectively. The 75% PC treatment had a relatively minor impact on seed production, showing resulting in 228 seeds/pod compared to 242.2 seeds/pod in the control. Similarly, seed weight exhibited a clear decline under drought stress conditions. Seeds from the control had the highest weight (i.e., 12.3 mg per 100 seeds), followed by the 75% PC, 50% PC, and 25% PC treatments, which weighed 10.5 mg, 8.5 mg, and 7.2 mg per 100 seeds, respectively. Typical seed appearances are shown in Supplementary Figure S3.

### Progeny drought stress memory

**Figure 4:**
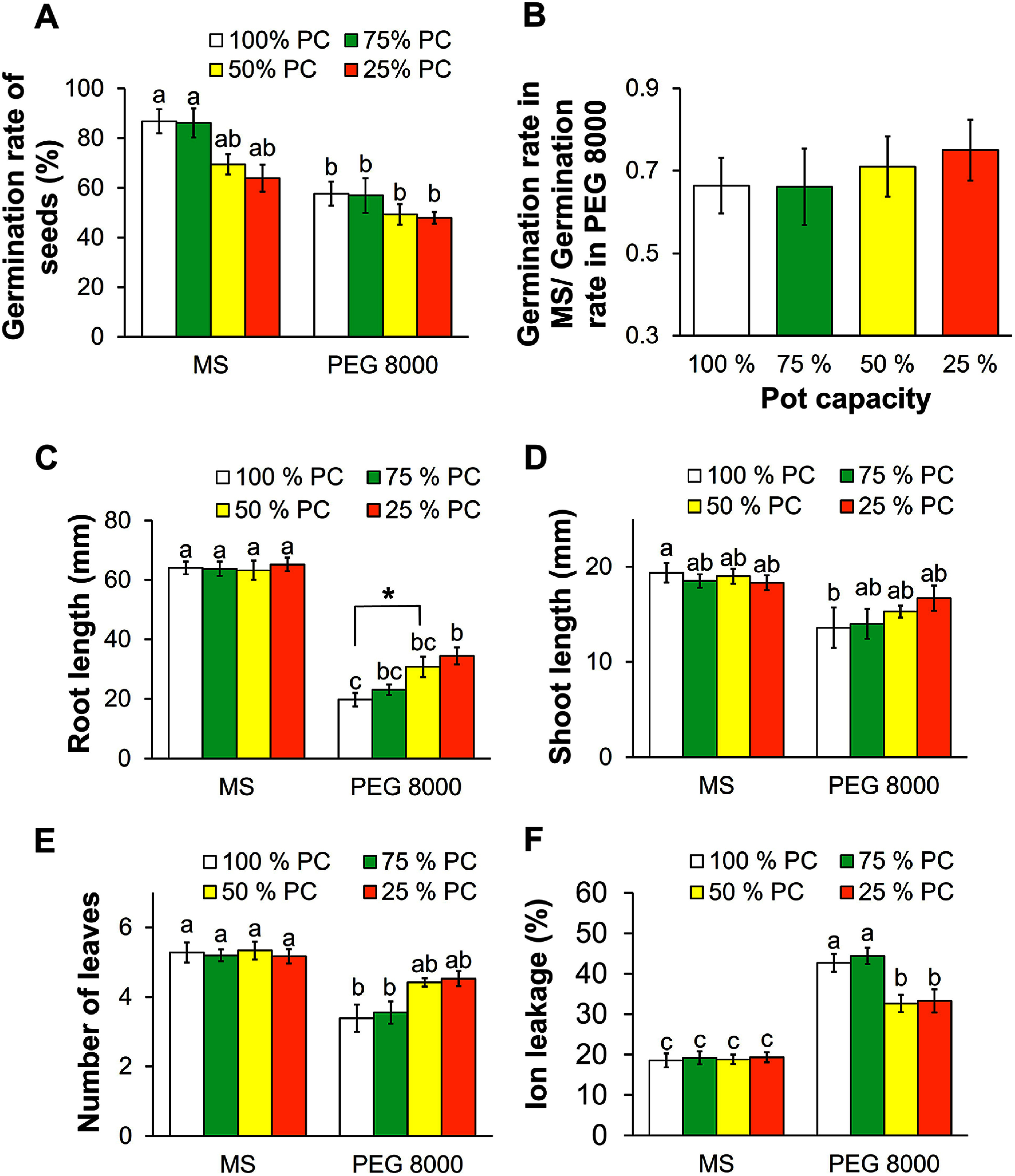
Figure 4. Effects of dehydration stress on seed germination rate, seedling vigor of offspring, and ion leakage. (A) Seed germination rate (four replicates of 36 seeds each). (B) The ratio of seed germination rates in the MS and PEG 8000 media. (C) Root length of seedlings, (D) Shoot length of seedlings, (E) Number of leaves of seedlings, and (F) Ion leakage of seedling leaves from the four parental stress treatments after 14 days in dehydration medium (four replications of nine seedlings each). Different letters indicate significant differences among treatments identified by Tukey-HSD tests (*p*<0.05). The asterisk signs denote significant differences between the control (100% PC) and 50% PC treatments identified by Student’s *t*-tests; Significance level: * *p*<0.05. Error bar: Standard error.

Seed germination rates in a control medium suggested a negative impact on seed quality due to parental drought levels. For example, seed germination rates were around 86% for both the 100% PC and 75% PC treatments, but this significantly reduced to 69.4% and 63.9% for the 50% PC and 25% PC treatments, respectively (Figure 4A). However, in the dehydration medium we observed no significant differences in germination rates among the different parental stress levels, with rates ranging from 47.9% to 57.4% (Figure 4A, Supplementary Figure S4). Next, calculating the ratio between the germination rate in the PEG 8000 medium and the corresponding rate in the MS medium revealed an interesting pattern. This ratio was approximately 0.66 for both the 100% PC and 75% PC treatments but increased to 0.71 and 0.75 for the 50% PC and 25% PC treatments (Figure 4B). Thus, it appears that seeds from parents that experienced moderate and severe stress exhibited higher tolerance to dehydration conditions.

Next, seedling vigor assessments were performed. Here, when germinated seedlings with similar (i.e., 1 mm) hypocotyl lengths were selected, no differences were observed among seedlings from the four different parental stress levels when grown in MS medium with respect to root length, shoot length, or the number of leaves (Figure 4C–E). However, in the dehydration medium, the enhanced drought tolerance of seedlings from parents exposed to moderate and severe stress was clearly evident. Seedlings from the 50% PC and 25% PC treatments exhibited longer roots, longer shoots, and more leaves relative to those from the 100% PC and 75% PC treatments. Of all seedlings, those from the 25% PC treatment displayed the longest roots (34.4 mm), the longest shoots (16.7 mm), and the highest number of leaves (4.5). Seedling vigor in PEG 8000 is shown in Supplementary Figure S5. Furthermore, under control conditions, the IL results of seedlings ranged from 18.6 to 19.3, revealing no significant differences among parental treatments. However, in the PEG 8000 medium, seedlings from the 100% PC and 75% PC treatments exhibited significantly higher damage levels relative to those from the 50% PC and 25% PC treatments, ranging from 42.7% to 44.4% and 32.7% to 33.3%, respectively (Figure 4F). Taken together, these results indicate that the memory of drought stress was transmitted to progeny.

## Discussion

### Effects of water deficit on vegetative traits

Decreased soil humidity (Supplementary Figure S1) resulted in reduced water uptake by roots, causing a decline in leaf turgor and triggering stomatal closure, which minimizes water loss through evaporation. Stomatal regulation was mediated by abscisic acid (ABA), which plays a crucial role in prompt responses to water scarcity (Liu et al. 2022b). During periods of prolonged drought stress, ABA is known to trigger the synthesis and accumulation of osmoprotectants to preserve membrane integrity and safeguard the photosynthetic apparatus (Hussain et al. 2021). Our study evaluated stomatal aperture during the final week of the stress period, and revealed a reduction in stomata and stomatal pore size in samples exposed to the 50% PC and 25% PC treatments (Figure 1D). This response was indicative of a long-term adaptation to drought stress and had a detrimental impact on photosynthetic efficiency (Figure 1A, B) and growth rate (Figure 1C).

Next, Fv/Fm values demonstrated a negative correlation with the severity of drought stress, as illustrated in Figure 1B. For example, Fv/Fm values below 0.78 are considered indicative of stress in plants, making photosynthesis a valuable parameter for selecting ornamental plants with drought tolerance (Toscano et al. 2019). We found that Chl values increased during the initial stages of light and moderate stress but decreased in response to prolonged stress or under severe conditions (Figure 1A). Similar results have been observed in previous studies (Hasanuzzaman et al. 2017; Zhu et al. 2020). Increases in Chl values may be due to decreased leaf area and increased leaf thickness (Handayani and Watanabe 2020). However, in response to long-term drought stress, chlorophyll degradation occurs more seriously, and can lead to reductions in Chl (Damour et al. 2009). Similarly, RGR responded in accordance with the severity of stress (Figure 1C). Drought stress can disrupt the photosynthesis system and can result in reduced mineral nutrient uptake. The decrease in energy and loss of turgor pressure inhibited cell expansion and cell division, ultimately reducing RGR (Yang et al. 2021).

The response of *Petunia* plants to the severity of drought stress was characterized by a decrease in RWC and an increase in IL. RWC serves as a widely used indicator for assessing drought stress, since the balance between leaf tissue water supply and transpiration rate is reflected by the RWC metric (Soltys-Kalina et al. 2016). As RWC decreases, it leads to stomatal closure, resulting in elevated leaf temperatures and reduced plasma membrane flexibility (Seleiman et al. 2021). Moreover, during long periods of drought stress, ABA triggers the synthesis and accumulation of osmolytes to maintain cellular structural stability (Takahashi et al. 2020). However, our study revealed a notable vulnerability of cell membranes when the IL dramatically increased in the 25% PC treatment (Figure 1F). This observation suggested that osmoprotectants had not accumulated to sufficient levels for effective osmotic adjustment to drought stress.

### Effect of drought stress on flowering time

Reproductive stage has been shown to be highly sensitive to drought stress (Oguz et al. 2022). Here, the FBT and the FFT were both significantly influenced by stress, but were influenced by moderate (50% PC) and severe stress (25% PC) in different ways. For example, the 50% PC treatment resulted in delayed bud formation and flowering, while the 25% PC treatment led to earlier flowering (Figure 2A). These observations suggested that *Petunia* plants exposed to moderate and severe stress employ different strategies to respond to drought stress. Under moderate drought, plants adopted a drought avoidance strategy; that is, they tended to delay flowering and reduce RGR as a form of adaptation to limited energy availability. In other studies, plants adopting this strategy showed reduced water loss through transpiration and minimal metabolic activity (Chirivì and Betti 2023; Toscano et al. 2019).

Typically, the transition to the plant’s reproductive stage is governed by floral genes under the regulation of photoperiodic factors. Three key regulatory genes involved in photoperiodic control are *GIGANTEA* (*GI*), *CONSTANS* (*CO*), and *FLOWERING LOCUS T* (*FT*). These genes, identified in *Arabidopsis*, are expressed in the vascular tissue of leaves (Andrés and Coupland 2012). The gene *FT* encodes a protein that plays a central role in the regulation of flowering (Pin and Nilsson 2012). Drought escape (DE) occurs when early flowering is triggered in response to water scarcity, and serves as a survival strategy to complete the life cycle and overcome unfavorable environmental conditions (Franks 2011; Sivakumar and Srividhya 2016). In general, the *GI* gene is present only as a single copy in most plants, although in Solanaceae genomes, there can be two or three copies (Bombarely et al. 2016). As a member of the Solanaceae family, the *Petunia* genome possesses two orthologous *GI* genes, known as *PhGI1* and *PhGI2* (Brandoli et al. 2020). When *PhGI1* was overexpressed in *Petunia* plants, it was found to inhibit vegetative growth, promote flower formation, and prevent bud shading, but did not appear to play a role in the transition to the reproductive stage. The function of *PhGI2* has not yet been discovered, but it is anticipated to play a role in flowering time (Brandoli et al. 2020).

In this study, *Petunia*, a facultative long-day plant for flowering (Warner 2010), was cultivated in a tropical region with a neutral day length of 11.5 to 12.5 h of daylight. Notably, early flowering was observed under severe drought stress, occurring 5.3 days earlier than in the control group. This phenomenon indicates a potential mechanism whereby the accumulation of endogenous ABA at high levels, induced by severe drought stress, may activate the expression of *PhGI*. This in turn upregulates *FT-like* genes and ultimately enhances the flowering process. A study on *Petunia* has previously shown that *PhFT* can induce flowering in both *Petunia* and *Arabidopsis* when overexpressed (Tsukamoto et al. 2016). In the case of *Arabidopsis*, which is considered a long-day flowering plant (Koornneef et al. 1991), under long-day conditions the GI protein upregulates the *CO* gene by interacting with other factors. Subsequently, the CO protein activates *FT* transcription (Andrés and Coupland 2012). However, under drought stress conditions that cause ABA to accumulate to high levels, ABA upregulates the *GI* gene and subsequently activates other florigen genes such as *CO* and *FT*. Consequently, this has been found to lead to early flowering under long-day conditions (Riboni et al. 2016). In contrast, rice is classified as a short-day plant (Yano et al. 2001). Flowering has been found to be induced under short-day conditions by the Heading date 3a protein (i.e., Hd3a, an FT ortholog), and drought stress is known to trigger early flowering in rice under short-day conditions (Du et al. 2018). Two pathways have been proposed for DE in rice: the ABA-dependent and the ABA-independent pathways. In the ABA-dependent pathway, an increase in endogenous ABA under drought stress may have a positive impact on early flowering. ABA has been found to be able to activate the transcription of rice *TIMING OF CAB EXPRESSION 1* (*OsTOC1*), which subsequently upregulates the expression of *OsHd3a* and ultimately leads to flowering (Du et al. 2018). In addition, the ABA-independent pathway involves key genes, such as the *MADS box* gene *OsMADS50* and the *Heading date 1* gene (*OsHd1*, a *CO* ortholog) that respond to signals activating the flowering pathway. These genes enhance the expression of *OsHd3a*, which promotes flowering (Du et al. 2018). In the context of *Petunia*, early flowering might be caused by the upregulation of *PhGI* or *MADS box* genes that promote the expression of *PhFT-like* genes. In a previous report, *PETUNIA FLOWERING GENE* (*PFG*), also identified as a *MADS box* gene, was found to contribute to the transition from the vegetative to reproductive phases, and functions as an inflorescence meristem identity gene (Immink et al. 1999).

Overall, drought stress induced early flowering is a survival strategy to complete the life cycle and overcome unfavorable environmental conditions (Franks 2011; Sivakumar and Srividhya 2016). DE has often been observed in crops like rice (Du et al. 2018; Kumar et al. 2023) and wheat (Shavrukov et al. 2017) as well as in *Arabidopsis* (Riboni et al. 2013). In this study, early flowering was, for the first time, observed in *P. hybrida* plants in the 25% PC treatment group. However, the detailed mechanism by which this occurs remains unclear, and requires further study.

### Impacts of water scarcity on pollen viability, pollen germination, and seed production

Water deficit can damage the meiotic phase of pollen mother cells, resulting in a significant reduction in pollen grain production (Yu et al. 2019). Here, a 1.3-fold decrease in the number of pollen grains per flower and the viable pollen rate of samples in the 25% PC treatment was observed compared to the control (Figure 3B, C). In addition, there was a reduction of 1.2 and 1.6 fold in the pollen germination rates of the 50% PC and 25% PC treatments, respectively, relative to the control (Figure 3E). Water stress during the reproductive stage often causes pollen sterility in crops due to disturbances in carbohydrate metabolism (Kokubun et al. 2001). The viability and germination rates of pollen can also be adversely affected due to decreased starch accumulation within pollen grains. Sterile pollen often exhibit minimal or no starch content (Jin et al. 2013). Furthermore, drought stress can also disrupt the balance between ABA and gibberellic acid (GA), two crucial hormones for anther development (Yu et al. 2019). The accumulation of ABA during drought stress is known to inhibit GA, leading to sterility (Aya et al. 2009). Here, in the 25% PC treatment we observed that the seed number per pod, the seed germination rate, and the weight of seeds decreased by approximately 2 times, 1.4 times, and 1.7 times respectively, compared to the control group (Figure 3E, F). This reduction can be attributed to water scarcity, which hampers seed production and seed quality by reducing the number of pollen grains released from the anthers and pollen viability (Ji et al. 2010). Moreover, drought during seed formation is also associated with reduced carbon assimilation, and results in smaller and lighter seeds of reduced quality (Wijewardana et al. 2019).

### Transgenerational effects on seed germination and seedling vigor of progeny

Transgenerational effects were clearly observed in seedling vigor, since the root lengths of seedlings from the moderate and severe stress treatments was significantly longer than those from the other treatments (Figure 4C, Supplementary Figure S5). Water scarcity had a significant impact on root development, inhibiting the growth of the main root and the expansion of the root system (Wasaya et al. 2018). Seedlings with better root systems, reflected by a longer root length, can more efficiently absorb water and nutrients to support the growth of the aboveground portion of the plant, resulting in longer shoots and more leaves (Figure 4C–E). Therefore, root length under dehydration stress conditions is generally positively correlated with drought tolerance (Kou et al. 2022). These results contrast with previous reports, which suggests that an optimal maternal environment produces progeny that are better adapted to abiotic stress due to higher seed quality (Nguyen et al. 2021). Although parental drought stress during reproduction may affect seed quality, shape, and composition, it can also enhance drought tolerance in the next generation. Similar findings have been reported in *Brassica napus* (Hatzig et al. 2018) durum wheat (Liu et al. 2021), *Amaranthus palmeri* (Matzrafi et al. 2021), and rice (Zheng et al. 2017).

The existence of a transgenerational effect was further evidenced by the stability of cell membranes in the leaves of seedlings derived from parents subjected to the 50% PC and 25% PC treatments when placed under dehydration stress (Figure 4F). This stability is likely caused by the accumulation of osmoprotectants such as proline and glycine betaine (Wang et al. 2018). Increases in ABA levels during parental drought stress may be linked to the activation of transcription factors that regulate stress responses, heat shock protein genes, or chaperones involved in protein folding in maternal plants exposed to terminal drought. These factors, in some combination, likely assisted progeny in developing stress tolerance under drought conditions by maintaining better tissue water status (Tabassum et al. 2018). In another report, small RNAs were found to be associated with the transgenerational effects of drought stress (Liu et al. 2021). These effects represented a short-term memory mediated by morphological acclimation, physiological changes, as well as molecular and metabolic alterations (Crisp et al. 2016). Furthermore, drought stress memory can act as a long-term mechanism related to epigenetic processes, since it involves chromatin marks such as DNA methylation and histone modifications (X Liu et al. 2022). Parental drought stress memory can trigger more efficient and rapid stress responses in offspring, enabling them to adapt better to drought stress and recover more quickly (Aswathi et al. 2022). However, drought stress memory is not a universally applicable strategy, since its establishment depends on species and genotype (Jacques et al. 2021).

In conclusion, in this study we found that moderate drought stress (50% PC) delayed flowering time, while severe drought stress (25% PC) induced early flowering, a phenomenon that were observed in *P. hybrida* for the first time. To gain a clearer understanding of this early flowering phenomenon in *Petunia*, further research is needed to explore the functions of *PhGI2*, *PhFT-like* genes, and the signaling pathways involving *GI* genes and *FT* genes, including the potential involvement of both ABA-dependent and ABA-independent pathways. In addition, this study observed positive transgenerational effects induced by moderate and severe drought stress, as was evident in the vigor responses of seedlings and the decrease in IL of seedling leaves when subjected to dehydration stress. While parental drought stress during the reproductive stage may alter seed quality, shape, and composition, it can also enhance drought tolerance in the next generation. Consequently, a balance between potential decreases in seed quality due to parental stress and improved seedling vigor and stress tolerance in offspring must be carefully managed (Liu et al. 2022a). In this study, transgenerational memory of drought was unrelated to flowering time in the parental generation, but its effect in the offspring generation remained unknown. However, as these findings are preliminary, further research is necessary to investigate drought stress tolerance at different stages of development, both in the first generation and across multiple generations. It is also essential to explore cross-stress tolerance of progeny under various abiotic stress conditions, including heat and salt stress, to assess the interaction of genes involved in epigenetic processes.

## Abbreviations

ABA: abscisic acid
ANOVA: Analyses of Variance
C1: initial conductivity
C2: final conductivity
Chl: chlorophyll index
CO: constans
Co., Ltd.: Company Limited
DE: drought escape
DNA: deoxyribonucleic acid
DW: dry weight
FBT: first bud time
FFT: first flowering time
FT: Flowering locus T
Fv/Fm: the maximum quantum efficiency of photosystem II
FW: fresh weight
GA: gibberellic acid
GI: gigantea
H1: plant height at Time 1 (T1)
H2: plant height at Time 2 (T2)
Hd: Heading date
HSD: Tukey’s Honestly Significant Difference
i.e.: id est
IL: ion leakage rate
MS: Murashige and Skoog medium
P: probability
PC: pot capacity
PEG: polyethylene glycol
*PFG*: *Petunia flowering* gene
RGR: relative growth rate
RWC: relative water content
TOC1: Timing of cab expression 1
TW: turgid weight
USDA: United States Department of Agriculture

## Description of Supplementary Files

Supplementary Figure S1. Cultivation conditions during drought treatment time.

Supplementary Figure S2. Photos of typical plants before and after drought stress treatment.

Supplementary Figure S3. Photos of typical seeds from plants in different drought stress treatments.

Supplementary Figure S4. Photos of seeds’ germination in dehydration medium.

Supplementary Figure S5. Photos of seedlings’ development in dehydration medium.

Supplementary Table S1. Timing of recording indicators in the drought stress assay.
