# Supplementary material for: Drought stress induces early flowering and the stress tolerance of offspring in Petunia hybrida
Source: Plant Biotechnol (Tokyo). 2024 Mar 25;41(1):53–63. doi: 10.5511/plantbiotechnology.23.1220a (PMC11500584; doi:10.5511/plantbiotechnology.23.1220a)
Supplement: Supplementary Data [file plantbiotechnology-41-1-23.1220a-s001.pdf]

Supplementary Files

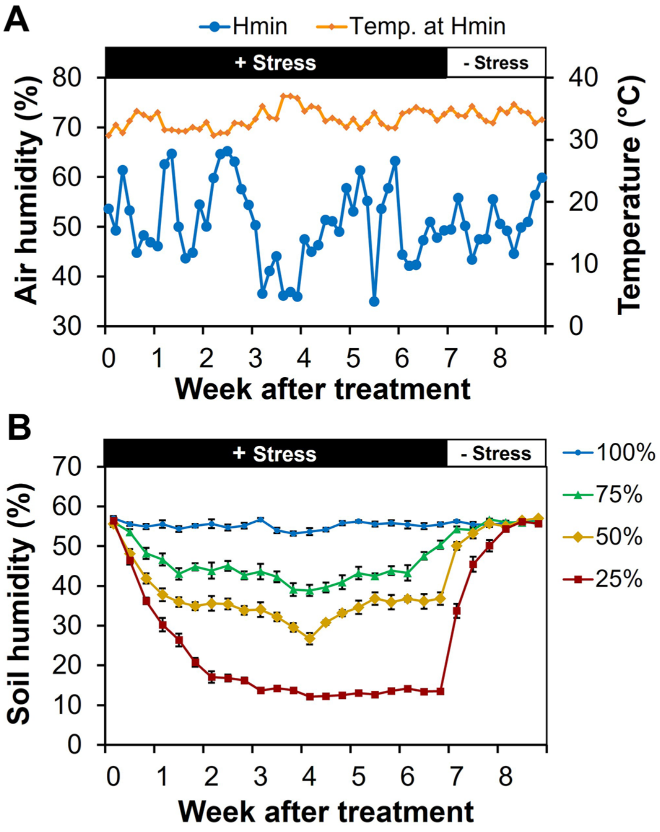

Supplementary Figure S1. Cultivation conditions during drought treatment time. (A) Air humidity and air temperature in the greenhouse; (B) Soil humidity of plant pots. Error bar: Standard error. "+Stress" indicates the Drought stress period (7 weeks) with four different irrigation levels (100% PC, 75 % PC, 50 % PC, and 25 % PC); "—Stress" indicates the Recovery period (2 weeks), after Drought stress, with 100% PC in irrigation levels.

Air humidity, air temperature, and soil humidity were recorded in a greenhouse located in Thu Duc City, Ho Chi Minh City, Vietnam, from October 2021 to February 2022. This timeframe offered favorable temperature and humidity conditions for *Petunia* cultivation. The greenhouse experienced its lowest air humidity levels around 2:00 p.m. in a day. This drop in air humidity significantly influenced water evaporation within the soil pots, thereby strongly impacting soil humidity levels.

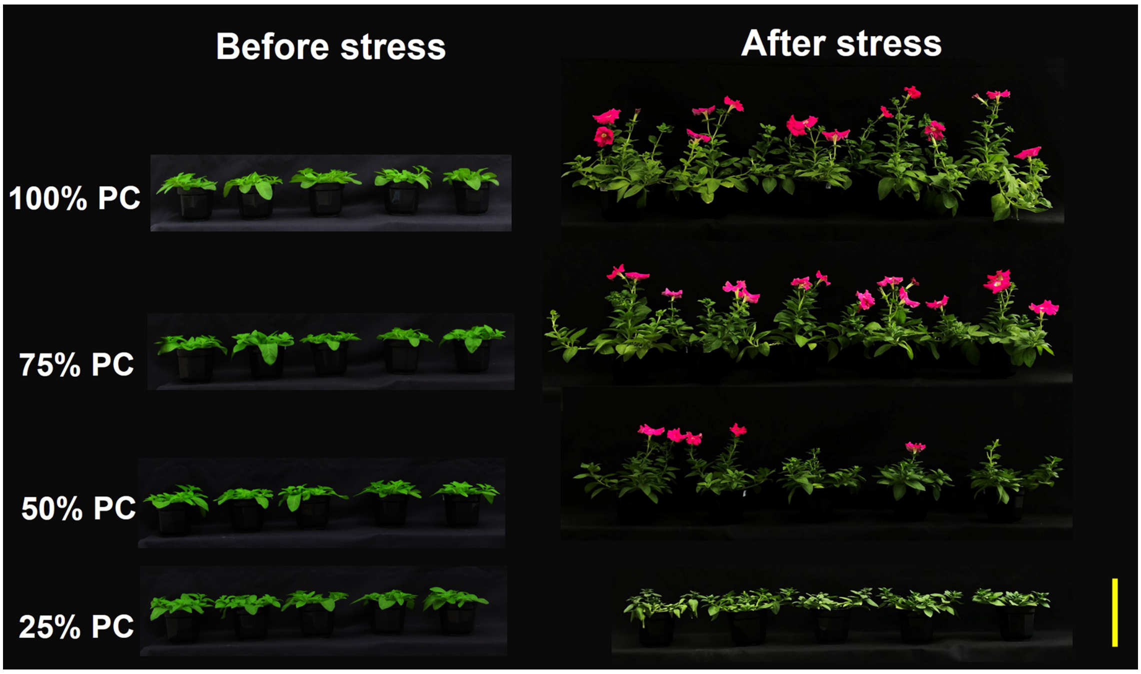

Supplementary Figure S2. Photos of typical plants before and after drought stress treatment. Scale bar: 20 cm. Before stress photographs were taken on the first day of the 6-week drought stress treatments to the 9-week-old plants. After stress photographs were taken on the last day of week 6.

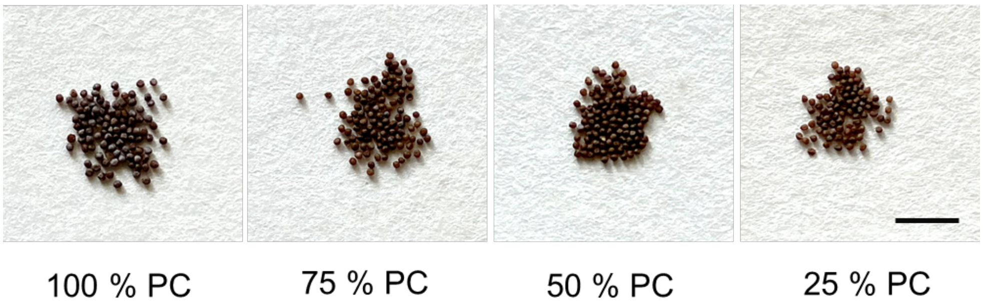

Supplementary Figure S3. Photos of typical seeds from plants in different drought stress treatments.

Scale bar: 5 mm

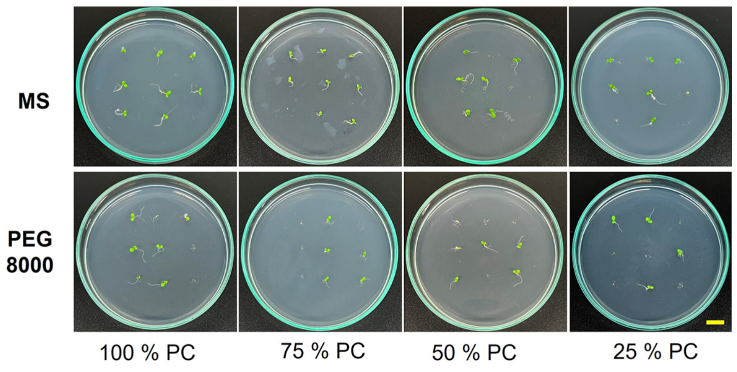

Supplementary Figure S4. Photos of seeds' germination in dehydration medium. Seeds were from plants in different drought stress treatments. Scale bar: 1 cm

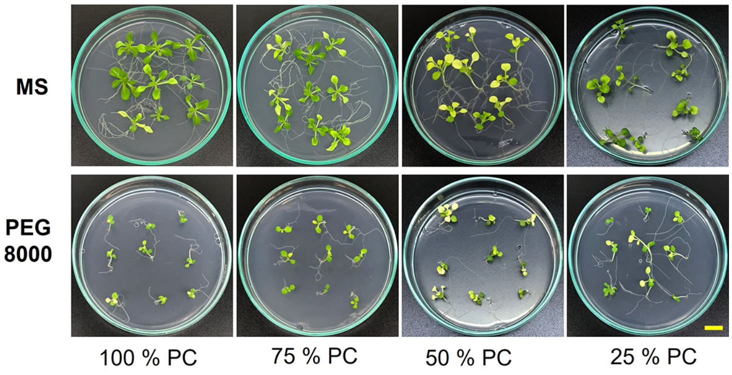

Supplementary Figure S5. Photos of seedlings' development in dehydration medium. Seeds were from plants in different drought stress treatments. Scale bar: 1 cm

Supplementary Table S1. Timing of recording indicators in the drought stress assay

Treatments	Week	Soil humidity (%)	Chl + Fv/Fm	Plant height (mm)	Ion leakage rate (%)	Relative water content (%)	Stomatal trails	Pollen quantification and viability	Pollens germination	Harvest seeds	Taking photo	Time of first bud/ first flower in each pot
**Drought stress**<br>4 treatments<br>- Control: 100 % PC<br>- Water deficit treatment: 75 % PC; 50 % PC; 25 % PC	**0** (Initial)	x	x	x							x (first day of this week)	Record when it happens
	**1**	x										
	**2**	x	x	x								
	**3**	x										
	**4**	x	x	x				x	x			
	**5**	x										
	**6**	x	x	x	x	x	x				x (last day of this week)	
**Recovery** (100% PC)	**7**	x										
	**8**	x	x	x								
